# The Novel Phages phiCD5763 and phiCD2955 Represent Two Groups of Big Plasmidial Siphoviridae Phages of *Clostridium difficile*

**DOI:** 10.3389/fmicb.2018.00026

**Published:** 2018-01-22

**Authors:** Gabriel Ramírez-Vargas, Shan Goh, César Rodríguez

**Affiliations:** ^1^Facultad de Microbiología and Centro de Investigación en Enfermedades Tropicales, Universidad de Costa Rica, San José, Costa Rica; ^2^Pathobiology and Population Studies, Royal Veterinary College, Hatfield, United Kingdom

**Keywords:** *Clostridium difficile*, big bacteriophages, siphovirus, phiCD5763, phiCD2955, phiCD211/phiCDIF1296T

## Abstract

Until recently, *Clostridium difficile* phages were limited to Myoviruses and Siphoviruses of medium genome length (32–57 kb). Here we report the finding of phiCD5763, a Siphovirus with a large extrachromosomal circular genome (132.5 kb, 172 ORFs) and a large capsid (205.6 ± 25.6 nm in diameter) infecting MLST Clade 1 strains of *C. difficile*. Two subgroups of big phage genomes similar to phiCD5763 were identified in 32 NAP_CR1_/RT012/ST-54 *C. difficile* isolates from Costa Rica and in whole genome sequences (WGS) of 41 *C. difficile* isolates of Clades 1, 2, 3, and 4 from Canada, USA, UK, Belgium, Iraq, and China. Through comparative genomics we discovered another putative big phage genome in a non-NAP_CR1_ isolate from Costa Rica, phiCD2955, which represents other big phage genomes found in 130 WGS of MLST Clade 1 and 2 isolates from Canada, USA, Hungary, France, Austria, and UK. phiCD2955 (131.6 kb, 172 ORFs) is related to a previously reported *C. difficile* phage genome, phiCD211/phiCDIF1296T. Detailed genome analyses of phiCD5763, phiCD2955, phiCD211/phiCDIF1296T, and seven other putative *C. difficile* big phage genome sequences of 131–136 kb reconstructed from publicly available WGS revealed a modular gene organization and high levels of sequence heterogeneity at several hotspots, suggesting that these genomes correspond to biological entities undergoing recombination. Compared to other *C. difficile* phages, these big phages have unique predicted terminase, capsid, portal, neck and tail proteins, receptor binding proteins (RBPs), recombinases, resolvases, primases, helicases, ligases, and hypothetical proteins. Moreover, their predicted gene load suggests a complex regulation of both phage and host functions. Overall, our results indicate that the prevalence of *C. difficile* big bacteriophages is more widespread than realized and open new avenues of research aiming to decipher how these viral elements influence the biology of this emerging pathogen.

## Introduction

*Clostridium difficile* is a Gram-positive, rod-shaped, spore-forming, anaerobic bacterium that can cause a variety of diseases in humans and some animal species. It is the most common cause of hospital acquired diarrhea (Vindigni and Surawicz, 2015) and, under some circumstances, *C. difficile* infections (CDI) may develop into life-threatening conditions such as fulminant colitis or toxic megacolon. This pathogen primarily affects elderly people with comorbidities, patients undergoing surgery, long-term hospitalized patients, and immuno-compromised patients. However, the epidemiology of CDI changes over time in groups at risk of infection and in dominant strains (Goudarzi et al., 2014), such that now community-acquired CDI is more prevalent and the “hypervirulent” epidemic NAP1/RT027 strains are no longer predominant. Instead, the NAP7/RT078 or NAP9/RT017 strains are emerging among humans and animals from Asia, Australia, Costa Rica (López-Ureña et al., 2016) and other regions of the world (Janoir, 2016). Although reasons for this change are unclear, phage and other mobile genetic elements (MGE) are thought to contribute to the evolution of *C. difficile*, as it has been demonstrated for other human pathogens (Davies et al., 2016).

The interactions between *C. difficile* and phage seem to be frequent and biologically relevant. In this regard, multiple CRISPR arrays are present in most sequenced *C. difficile* genomes (Andersen et al., 2016), and the pathogenicity locus harboring toxin genes is likely derived from temperate bacteriophages (Janoir, 2016). In addition, *C. difficile* prophages have been shown to modulate toxin production (Dingle et al., 2014) or carry *agr*-like quorum sensing systems that regulate various virulence factors (Hargreaves et al., 2014).

With nearly 6,000 representatives, the double-stranded DNA tailed bacteriophages are the largest viral group known to date. They are classified in the order Caudovirales, which is divided into three families: the Myoviridae with long contractile tails, the Siphoviridae with long non-contractile tails, and the Podoviridae with short tails. Phage genomes 30–75 kb are known as medium phages (Casjens, 2005), those with genomes between 130–139 kb are termed big phages, and phage with genomes >200 kb are known as jumbo or giant phages (Hendrix, 2009). Nearly all *C. difficile* phage described to date belong to the *Myoviridae* or *Siphoviridae* families and have genomes of medium size (32–57 kb). A *C. difficile* phage genome of 131 kb termed phiCD211 was deposited in public databases by Monot and collaborators in 2014 (GenBank accession number: NC_029048.1). The same phage genome was independently published by Wittmann et al. (2015) and named phiCDIF1296T (GenBank accession number: CP011970.1). phiCD211/phiCDIF1296T is of great interest due to the size of its genome and novel genome features such as multiple transposases and regions of bacterial origin, as well as several transcriptional regulators/DNA binding proteins.

A group of *C. difficile* strains classified as NAP_CR1_/RT012/ST-54 caused a serious outbreak of CDI in a large Costa Rican hospital in 2009 (Quesada-Gómez et al., 2010). These isolates belong to the MLST Clade 1, show increased virulence in cell and animal models (Quesada-Gómez et al., 2015), and are now endemic in Costa Rica hospitals (López-Ureña et al., 2016). Their genomes were found to be extremely diverse, as indicated by their distribution in at least 10 *Sma*I macrorestriction patterns, the small size of their core genome (74%), and the large number of MGE-associated genes in their genomes (Ramírez-Vargas et al., 2017). Without exception, we found large extrachromosomal circular contigs in WGS obtained for several NAP_CR1_/RT012/ST-54 isolates from different hospitals and time points, therefore this study aimed to determine whether these contigs were phage genomes and to investigate phage novelty by sequence comparison to other *C. difficile* phage genomes.

## Materials and methods

### Bacterial cultures

Thirty-two *C. difficile* NAP_CR1_ strains from stool samples positive for TcdB by rapid inmunochromatographic assays (Techlab) were used for whole genome sequencing (Supplementary Table 1). Six additional *C. difficile* strains from other sources were used only for phage assays (Supplementary Table 2). *C. difficile* was grown anaerobically in brain heart infusion broth (BHI) for 3–4, 6–8, or 16–20 h for phage assays, PFGE, and DNA extractions, respectively.

### PFGE typing

*Clostridium difficile* NAP_CR1_ strains were typed by PFGE as previously described (Quesada-Gómez et al., 2010). Briefly, agarose plugs were prepared by mixing equal volumes of bacteria and Seakem Gold agarose (Lonza) in 1X Tris-EDTA buffer containing SDS (Sigma). Embedded bacteria were lysed with lysozyme (Sigma) and mutanolysin (Sigma) and their DNA was digested with *Sma*I (Roche). The resulting DNA fragments were separated on 1% agarose gels (Bio-Rad) prepared with 0.5X Tris borate-EDTA buffer and 50 μM thiourea (Sigma) using a CHEF-DRII system (Bio-Rad). Digitalized images were analyzed with the BioNumerics software (version 6.0; Applied Maths).

### Whole genome sequencing and bioinformatic analyses

Bacterial genomic DNA was extracted using a commercial kit (DNEasy Blood & Tissue Kit, Qiagen). Draft-quality WGS for the 32 NAP_CR1_ isolates and a non-NAP_CR1_ isolate were obtained at the Sanger Institute by sequencing-by-synthesis on a HiSeq instrument (Illumina) using multiplexed paired-end libraries. The length of the reads was 100 bp and each run generated ca. 3 millions reads with an average of 56 × coverage. Sequence data is available from the European Nucleotide Archive (Supplementary Table 1). Reads were assembled using Velvet (Zerbino and Birneye, 2008) or Edena (Hernandez et al., 2008) and then mapped back to assembly contigs using BWA (Li and Durbin, 2009) or Bowtie (Langmead et al., 2009) to detect misassemblies. For automated annotation we used Prokka v. 1.11 (Seemann, 2014) and databases containing *C. difficile* genomes of reference strains. These annotations were refined manually using CDD (Marchler-Bauer et al., 2015), PRODOM (Bru et al., 2005), SMART (Letunic et al., 2015), Uniprot (UniProt Consortium, 2015), Interpro (Finn et al., 2017), RAST (Overbeek et al., 2014), Pfam (Finn et al., 2014), and Virfam (Lopes et al., 2014) searches. Putative phage genome sequences are available in the NCBI GenBank bioproject PRJNA395540.

To detect and compare publicly available big phage genome sequences, the genomes of phiCD5763 and phiCD2955 were used as queries in BLAST searches against *C. difficile* Whole Genome Shotgun projects. Reads from projects showing hits (Supplementary Table 3) were assembled and annotated, and candidate contigs were probed for circularity using apc (https://github.com/tseemann/apc).

Pairwise nucleotide and protein sequence comparisons were performed with BLAST to calculate maximum identity and coverage percentages. Homologous gene families from predicted proteomes were clustered with the OrthoMCl and COGtriangles algorithms using GET_HOMOLOGUES (Contreras-Moreira and Vinuesa, 2013). The presence/absence matrix that was derived from this analysis and a concatenated ClustalW (Larkin et al., 2007) alignment containing the predicted protein sequences for the putative terminases TerS and TerL, the polymerase Polα, and ParM were exploited to generate rooted parsimony and neighbor-joining trees, respectively. These four coding sequences (CDS) are scattered along the genomes, hence they were arbitrarily included in the analysis to attenuate the masking of phylogenetic signals by recombination events. Furthermore, terminase sequences are often used to reconstruct viral phylogenies. ClustalW was used to align and thereby compare the sequence of putative receptor binding proteins (RBPs) in the phage genomes.

To assess the level of heterogeneity of the reconstructed elements, Spine and AGEnt (Ozer et al., 2014) were used to delimitate core- and pan-genomes, and Roary (Page et al., 2015) was used to determine the number of genes associated with them. Phamerator was used to classify big phage coding genes into “phams,” which are families of related phage genes defined by the similarity of their predicted products (Cresawn et al., 2011). The organization of protein-encoding genes into phams reveals which genes are most prevalent in a dataset. To identify signature sequences among the big phage genomes, we repeated the Phamerator analyses with an enlarged dataset that included the genomes of the 22 *C. difficile* phage genomes of medium size (Supplementary Table 4).

All phylogenetic reconstructions were visualized with FigTree (http://tree.bio.ed.ac.uk/software/figtree/). Linear and circular comparison figures were generated with Easyfig (Sullivan et al., 2011) and BRIG (Alikhan et al., 2011), respectively.

### Phage induction and propagation

A 16–20 h culture of *C. difficile* LIBA-5763 in BHI broth (Oxoid) was induced with mitomycin C (3 μg/mL, Roche) and the supernatant was filtered through a 0.22 μm membrane filter. This filtrate was tested for plaque production on six *C. difficile* isolates from human and animal sources (Supplementary Table 2). Log (3–4 h) and stationary phase (18–20 h) cultures were used as previously described (Goh et al., 2005). Single plaques were propagated on susceptible host isolates to obtain crude phage suspensions of ~1 × 10^9^ PFU/ml, which were semi-purified for electron microscopy and DNA extraction (Goh et al., 2005). Semi purification of phage involved removal of contaminating host nucleic acid by digestion with DNase I (100 μg/mL, Thermo Scientific) and RNase A (100 μg/mL, Roche) at 37°C for 30 min, overnight concentration of phage particles with PEG 6000 (10% w/v, Sigma), and 1 M NaCl (Sigma) at 4°C, and release of phage from PEG 6000 with 1 M KCl (Sigma). Phage particles were resuspended in phage buffer [0.15 M NaCl, 10 mM Tris (pH 6.5), 10 mM MgSO_4_, 1 mM CaCl_2_]_._

### Transmission electron microscopy

Purified phage suspensions were placed on the top of carbon film fixed on copper disks (Sigma) Excess solution was removed, the grids were washed with distilled water, and negatively stained with 2% uranyl acetate. Pictures were taken with a transmission electron microscope (Hitachi 7100) at magnifications of 30,000X and 60,000X at 100 kV.

### PCR-based detection of phage DNA

Phage DNA was extracted from semi-purified phage suspensions using the High Pure Viral Nucleic Acid Kit (Roche) and *C. difficile* genomic DNA was extracted from strains LIBA-5763 and CD630 using the DNeasy Blood and Tissue kit (Qiagen) for PCR. Primers were designed to target 479 bases of a gene predicted to encode a DNA sulfur protein, which is unique to phiCD5763–like phages. PCR was carried out with primers *dndB*-F (5′-TCTCTCATAACTCTGCTCCA-3′) and *dndB*-R (5′-AACTTGCACGAAACTCTTTC-3′), and Thermo Scientific PCR Master Mix (Fermentas). The cycling conditions included an initial denaturing step at 94°C for 5 min that was followed by 35 cycles at 94°C for 0.5 min, 1.5 min at 50°C and 1 min at 72°C, and a final elongation step for 7 min at 72°C.

## Results

### The genomes of several NAP_CR1_ isolates include large circular contigs that partially resemble phiCD211/phiCDIF1296T

A scrutiny of novel MGE in WGS of 32 NAP_CR1_ isolates consistently revealed the occurrence of circular contigs of 132 or 134 kb in different permutations. The 132 kb contigs were restricted to two isolates from the PFGE *Sma*I pattern 487 (LIBA-2945, LIBA-5763) and the 134 kb contigs were detected in NAP_CR1_ isolates from other nine *Sma*I patterns, including LIBA-5774 (Table 1). These circular contigs contained a large number of predicted CDS with similarity to hypothetical phage proteins, hence we hypothesized that they were phage genomes harbored in NAP_CR1_ isolates.

**Table 1:** Host and genome characteristics of two types of big phage found in Costa Rican isolates of *C. difficile*.

**Big phage**	**Host**					**Genome**			
	**Isolate(s)**	**Accesioned**	**Pulsotype**	**ST**	**Origin**	**Size (kb)**	**No. predicted ORF**	**%GC**	**tRNA**
phiCD5763^*^	LIBA-2945	2003	NAP_CR1_ PFGE-487	54	Human	132.5	172	26.3	Met (CAT) + Ser (GCT)
	LIBA-5763^*^	2009							
phiCD5774	30 NAP_CR1_ isolates	2003, 2009, 2011/2012	PFGE types 442, 447, 448, 449, 452, 488, 489, 558, and 578	54	Human	134.2	172	26.1	None detected
phiCD211/phiCDIF1296T	ATCC9689 = DSM1296^T^	1982	Unknown	3	Human	131.3	178/181	26.4	Ser (GCT)

* *The genome sequences of the big phage of isolates LIBA-2945 and LIBA-5763 are identical*.

**Figure 1:**
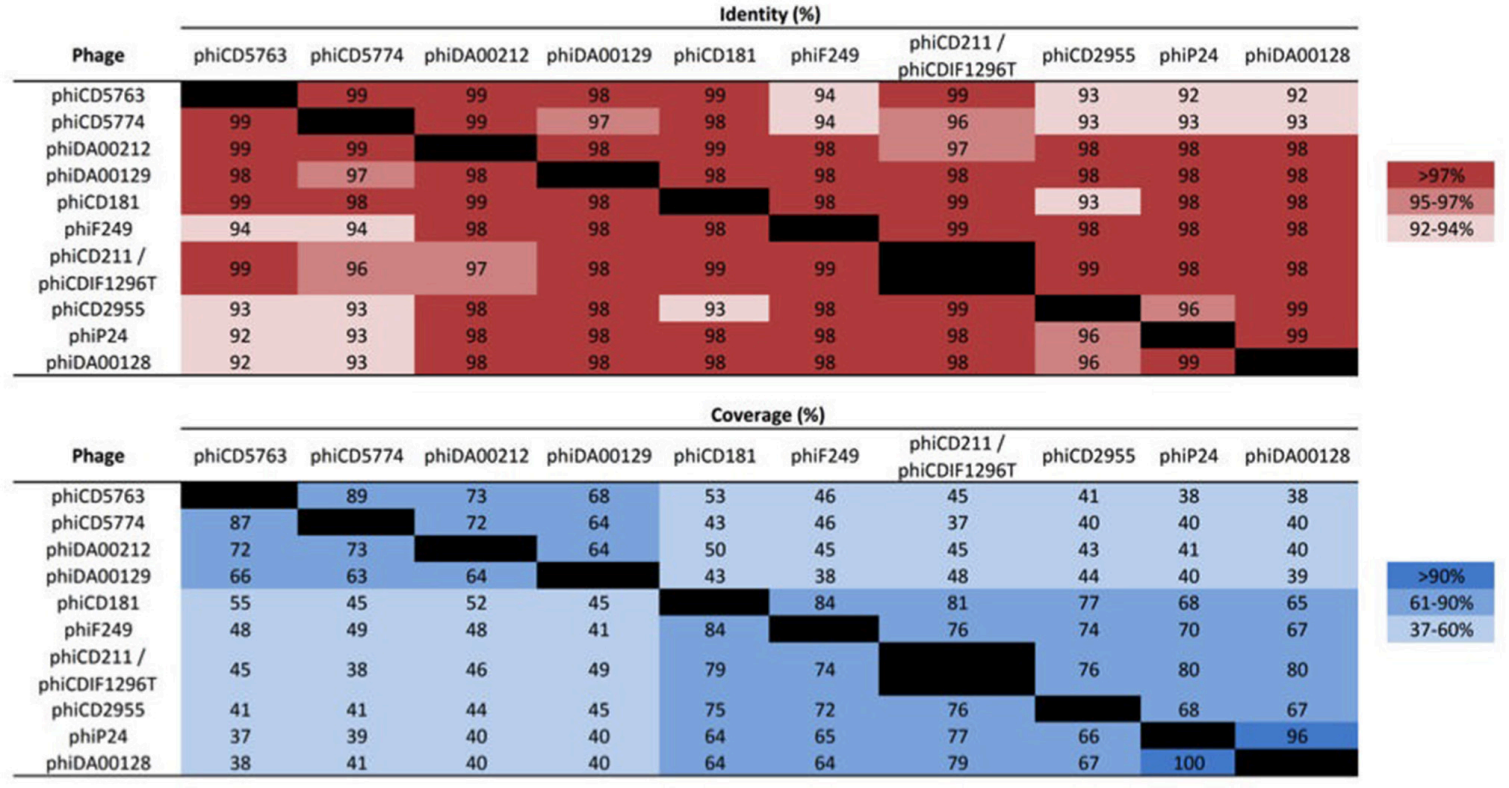
Nucleotide identity and coverage of big phage genomes. Whereas, the genomes of phiCD5763, phiCD5774, and phiCD2955 were assembled from reads generated in this study, the big phage genomes of isolates DA00212, DA00129, CD181, F249, P24/P25, and DA00128 were assembled from publicly available SRA data. The genomes of phiCD211/phiCDIF1296T were directly taken from GenBank. The corresponding accession numbers appear in the Supplementary Tables 1, 3. The big phage genomes of isolates P24 and P25 are identical.

The putative circular phage genomes of LIBA-5763 (phiCD5763, 132 kb) and LIBA-5774 (phiCD5774, 134 kb) partially resemble phiCD211/phiCDIF1296T with regards to their length (131–134 Kb), number and annotation of predicted ORFs (172–181), and GC-content (26%). Moreover, phiCD5763, but not phiCD5774, carry a tRNA–Ser gene, as already noted for phiCD211/phiCDIF1296T (Wittmann et al., 2015). Through Blast searches and pairwise alignments we determined that the phiCD5763, phiCD5774, and phiCD211/phiCDIF1296T genomes share 99–96% of nucleotide identity but only along 45–37% of their sequences (Figure 1). The proposed annotations of phiCD5763 and phiCD5774 are presented in Supplementary Material 1.

### Multiple *C. difficile* isolates carry a variety of circular extrachromosomal elements of ca. 130 kb

The partial sequence similarity of phiCD5763 and phiCD5774 to phage sequences reported in France (phiCD211) and Germany (phiCDIF1296T) prompted us to search for additional big phage genomes in genome databases. Forty-one isolates of human, bovine, and environmental origin from Canada, USA, UK, Belgium, Iraq and China had contigs matching the phiCD5763 genome (Supplementary Table 5). These isolates included representatives of 13 sequence types (ST) from MLST Clade 1, one ST from MLST Clade 2, one ST from MLST Clade 3, and one ST from MLST Clade 4 (Supplementary Table 5). Five isolates could not be fully classified because their genome sequences were highly fragmented, but they likely belong to the MLST Clade 1.

The non-NAP_CR1_ isolate from Costa Rica LIBA-2955 (ST49, PFGE *Sma*I pattern 479) was found to contain a distinct circular contig of 131 kb exhibiting a higher level of identity (99%) and coverage (76%) to phiCD211/phiCDIF1296T than the phiCD5763 and phiCD5774 genomes. We called this non-NAP_CR1_ phage phiCD2955 and its annotation is presented in Supplementary Material 2. Contigs covering the entire phiCD2955 sequence were found in 130 non-toxigenic or toxigenic *C. difficile* isolates of human or environmental origin recovered in Canada, USA, Hungary, France, Austria, and the UK (Supplementary Table 5). It was possible to allocate 118 of these isolates to 19 ST from MLST Clade 1 and two ST from Clade 2.

**Figure 2:**
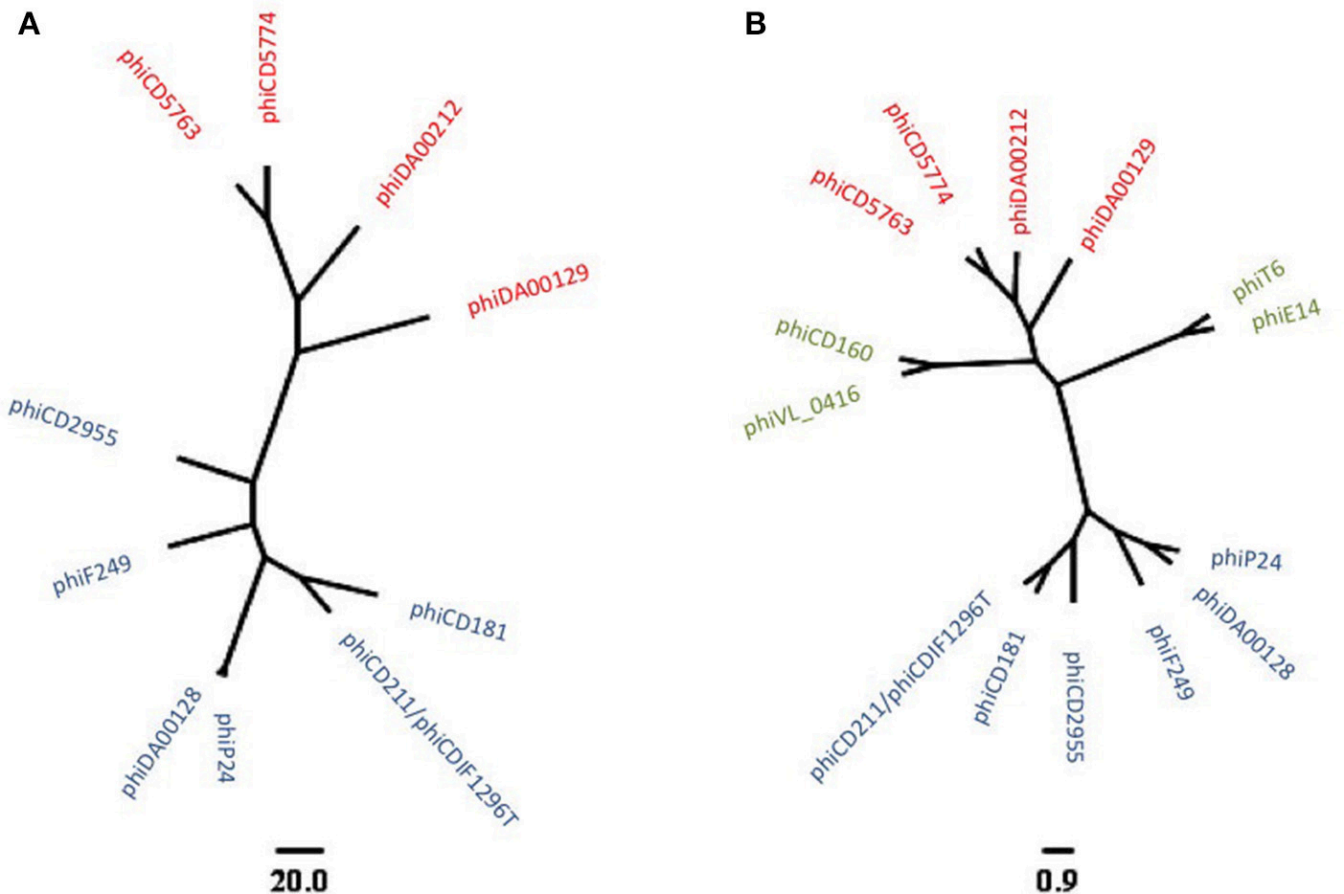
Phylogenetic relationship of ten reconstructed big phages genomes as revealed by predicted proteomes **(A)** or a concatenated alignment of TerS, TerL, Polα, and ParM protein sequences **(B)**. phiCD5763-like and phiCD2955-like elements appear in red and blue, respectively. An additional group of big phage with hybrid allele profiles became apparent after analysis of incomplete genome sequences (green in **B**). The scale bars correspond to number of gene cluster differences **(A)** or to the average number of amino acid substitutions per site **(B)**.

To reconstruct additional big phage genomes related to phiCD5763 and phiCD2955, we obtained all available Single Read Archives (SRA) files for the *C. difficile* isolates in Supplementary Table 5 and succeeded in assembling six full additional elements (phiDA00212, phiDA00129, phiCD181, phiF249, phiP24-25, phiDA00128) from the isolates DA00212, DA00129, P24, P25, DA00128, CD181, and F249 (Table 2). The proposed annotation of these additional putative phage genomes is presented in Supplementary Material 2. Based on length (128–135 kb), number of predicted ORFs (168–180), nucleotide identity (Figure 1) and gene content (Table 2), two and four of these reconstructed elements were classified as phiCD5763-like or phiCD2955-like phage, respectively. This classification was validated by phylogeny trees generated with the predicted proteome of all 10 genomes assembled (Figure 2A) and with the predicted protein sequences of two putative terminases (TerS, TerL), a DNA polymerase (Polα), and ParM (Figure 2B). Unexpectedly, four *C. difficile* isolates from the MLST clades 1 or C-I recovered in USA, Hungary, and Canada showed a hybrid pattern composed of a partial match to the TerS sequence and a full match to the TerL sequences of the phiCD5763-like phage genomes and the ParM and Polα sequences of phiCD2955 (Figure 2B, Supplementary Table 5). This hybrid pattern, along with the organization of their incomplete genomes (data not shown), suggests that a third group of big phage exists in *C. difficile*.

**Table 2:** Characteristics of phiCD5763-like and phiCD2955-like phage genomes reconstructed from WGS projects deposited in GenBank.

**Big phage**	**Isolate (s)**	**Phage type**	**Host**			**Genome**			
			**ST/MLST Clade**	**Origin**	**Year of isolation**	**Size (kb)**	**No. predicted ORF**	**% GC**	**tRNA**
phiDA00212	DA0212	phiCD5763-like	8/1	Human	2010	133.8	180	26.2	Ile (TAT) + Ser (GCT)
phiDA00129	DA00129		16?/1?	Human	2010	135.4	176	26.2	
phiCD2955	LIBA-2955	phiCD2955-like	49/1	Human	2009	131.6	172	26.5	Ser (GCT)
phiP24^*^	P24/P25		55/1	Human	2001	136.3	175	26.4	Ser (GCT)
phiDA00128	DA00128		3/1	Human	2010	131.9	170	26.4	Ser (GCT)
phiCD181	CD181		3/1	Human	2010	128.1	175	26.4	Ser (GCT)
phiF249	F249		63/1	Human	2010	128.3	168	26.4	None detected

* *The genome sequences of the big phage of isolates P24 and P25 are identical*.

### The phiCD5763- and phiCD2955-like genomes are modular

**Figure 3:**
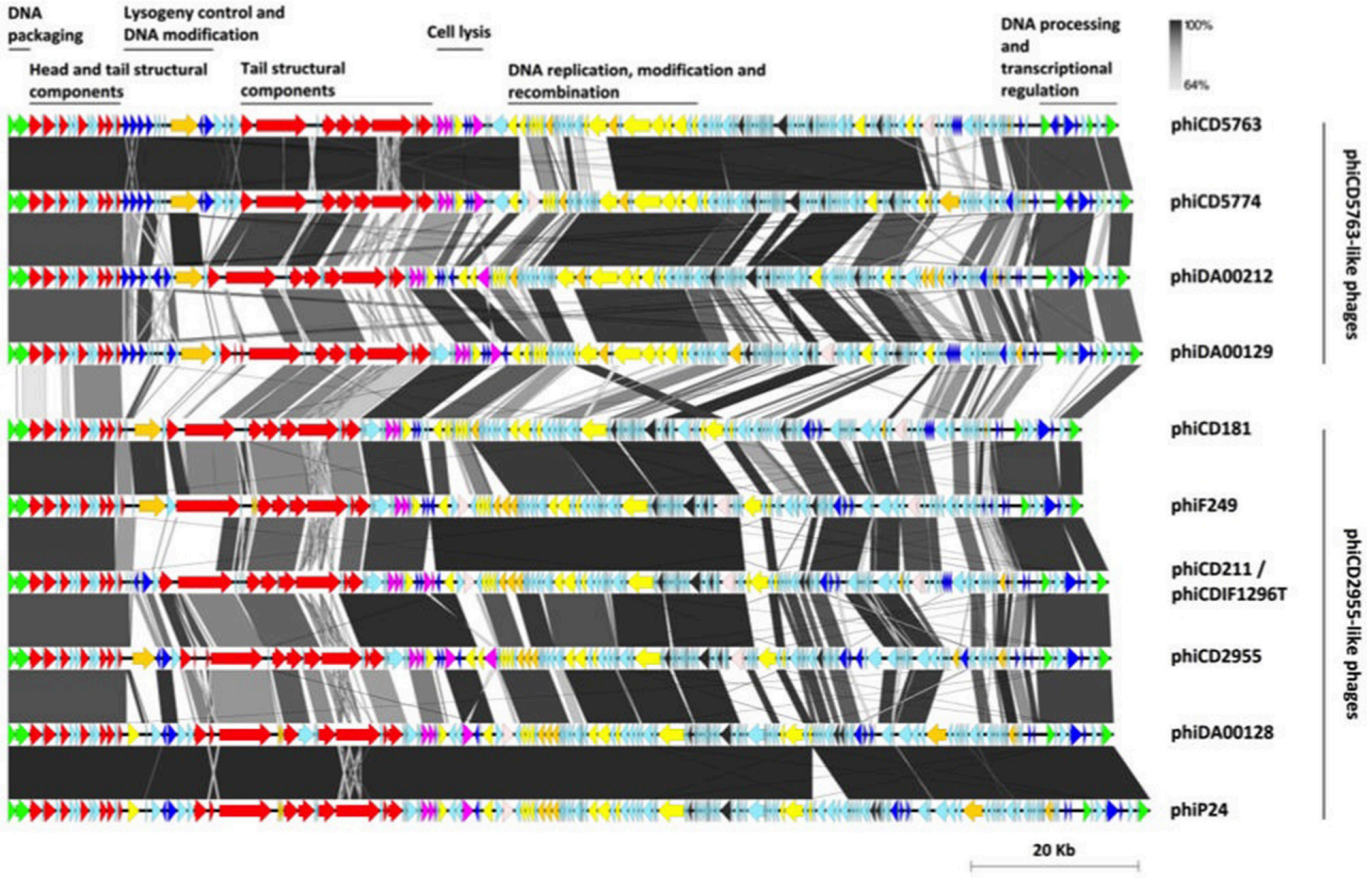
Modular organization of the phiCD5763-like and phiCD2955-like big phage genomes. The ORFs were color-coded according to their predicted functions: DNA processing (green), structural components (red), transcriptional regulation (navy blue), host adaptation (orange), lysis (purple), DNA replication and recombination (yellow), RNA metabolism (dark gray), transposases (light-pink), and hypothetical functions (light-blue). A bar whose length corresponds to 20 kb appears at the bottom right corner of the figure.

Although various genes encoding for hypothetical proteins are scattered along the reconstructed genome sequences, the proteins for which a function could be deduced showed a modular organization (Figure 3).

Following annotation convention, the phage genomes start with DNA packaging genes encoding the small and large subunits of a terminase complex and head morphogenesis genes, including a head closure protein. Tail associated genes were more numerous, including tail completion protein, neck protein (Ne1 homolog), a protein with a peptidase autocatalytic domain present in phage endosialidases, a RBP, and several tail fibers. This structural cluster was interrupted by a “lysogeny” control/transcriptional regulation and DNA modification module that includes transcriptional regulators, phage antirepressors and a DNA methylase. This regulation module was more clearly defined and contained more genes in the phiCD5763-like phage (5–7 annotated genes) than in phiCD2955-like phage (1–4 annotated genes).

A lytic-phase module followed with genes for putative amidases, an endolysin, a holin, and transcriptional regulators. The phiCD5763-like genomes have a DNA-replication-modification-recombination module with genes expected to encode a DNA polymerase III alpha chain, a ssDNA exonuclease, a DNA helicase, enzymes involved in nucleotide metabolism [a ribonucleotide reductase (RNR), a guanilate kinase, and a deoxyuridine 5′-triphosphate nucleotide hydrolase], a protein involved in DNA-sulfur modification, tyrosine recombinases, a Holliday junction resolvase, and a ERF-like protein. Most of these genes are also present in the phiCD2955-like phage, but in a more diffuse fashion.

Both groups of phage genomes harbored genes with homology to class II and III RNR enzymes. RNR enzymes are involved in nucleotide synthesis, hence important for DNA replication and repair (Nordlund and Reichard, 2006). Class II RNR is encoded by the *nrdJ* gene, and this system is oxygen independent hence found in facultative and strict anaerobes. Class III RNR is encoded by *nrdD* and *nrdG*, and are found only in strict anaerobes (Dwivedi et al., 2013). Phages phiCD2995, phiCD5774, phiF249, and phiDA00212 contained the class II RNR gene, while phages phiCD5763, phi24-25, phiDA00129, phiDA00128, phiCD181 contained class III RNR genes. Five out of six phiCD2955-like phages have three genes from a potential restriction/modification system involved in DNA processing.

The nine reconstructed big phage genomes and phiCD211/phiCDIF1296T continue with a large region composed mainly of hypothetical functions and genes for HTH type/phage antirepressors, a rRNA methyltransferase, a RNA 2′-phosphotransferase, a peptidyl-tRNA hydrolase, and tRNA-splicing ligase. Finally, all big phage genomes analyzed end with a module containing a LuxR-type transcription factor, a *parM*-like gene, and a calcineurin-like phosphatase.

A number of the phage genomes analyzed have genes encoding addiction mechanisms, such as a toxin/antitoxin system and a Fic/DOC protein, and two putative proteins annotated by RAST as a GPR-related spore protease and a PrpC-like serine/threonine phosphatase. The latter two genes were always found together in the same genomic location.

### Putative RBPs

The protein products of CD630_13740 (YP_001087872.1) and CDR20291_1218 (CBE03606.1) have been proposed as RBPs for diffocins present in various *C. difficile* chromosomes (Gebhart et al., 2012, 2015).

Proteins similar to those encoded by CD630_13740 and CDR20291_1218 were found in the tail modules of the phiCD5763, phiCD5774, phiCD2955, phiDA00212, phiDA00129, phiCD181, phiF249, phiP24, phiDA00128, and phiCD211/phiCDIF1296T genomes.

The putative RBPs of phiCD5763-like and phiCD2955-like phage were equally heterogeneous, as they shared 24–100 or 27–96% of identity, respectively. These RBPs could be distinguished in most cases. However, some phiCD2955-like phage RBPs (i.e., in phiDA00128, phiP24, and phiCD211/phiCDIF1296T) were more similar to RBPs of the phiCD5763-like phage phiDA00212, than to other phiCD2955-like phage RBPs (Supplementary Figure 1).

### The phiCD5763- and phiCD2955-like genomes are highly heterogeneous

Despite their modularity and proposed functional uniformity, the sequence heterogeneity of the putative big phage genomes was remarkable (Supplementary Material 3). Only 53% of nucleotides in the four phiCD5763-like genomes (71.2 kb) and 51–55% of nucleotides in the six phiCD2955-like genomes (70.2 kb) were shared by all members of each subgroup, and when all 10 phages were compared, only 20% (26.8 kb) of nucleotides were shared. Roary confirmed this finding, as only a low fraction of the genes was conserved in the phiCD5763-like phages (15%, 55/364), the phiCD2955-like phages (20%, 66/338), or all 10 big phage genomes (3%, 15/538).

Phamerator defined 328 phams from the 10 big phage genome assemblies (Supplementary Material 4). Sixty-six of these phams were omnipresent among phiCD5763-like and phiCD2955-like phage genomes (20%), but had distinct sequences. This universal group of phams includes structural proteins as well as proteins involved in replication, nucleotide metabolism, RNA processing, lysis, transcriptional regulation, and recombination (Supplementary Material 4).

When we sought phams unique to big phages through comparison of their genomes to those of medium size *C. difficile* phages, 60 out of 769 phams were detected in all 10 *C. difficile* complete big phage genomes (Supplementary Material 5). These signature phams include structural components (i.e., terminase, capsid, portal, neck, and tail proteins), the RBPs, proteins for DNA recombination and replication (recombinase, resolvase, primase, helicase, ligase, etc.), and many hypothetical proteins (Supplementary Material 5). Twenty of these 769 phams were unique among the phiCD5763-like big phages genomes, including proteins for phage antirepressors, a polymerase, a DNA-sulfur modification-associated protein, a tyrosine recombinase, ParM, transcriptional regulators, a DNA-binding protein, and several hypothetical proteins (Supplementary Material 5). The phiCD2955-like big phages genomes also had unique phams, but they were scarcer (*n* = 15). In this case, the differential phams include a DNA polymerase, ParM, a DNA-binding protein, and 12 hypothetical proteins (Supplementary Material 5).

**Figure 4:**
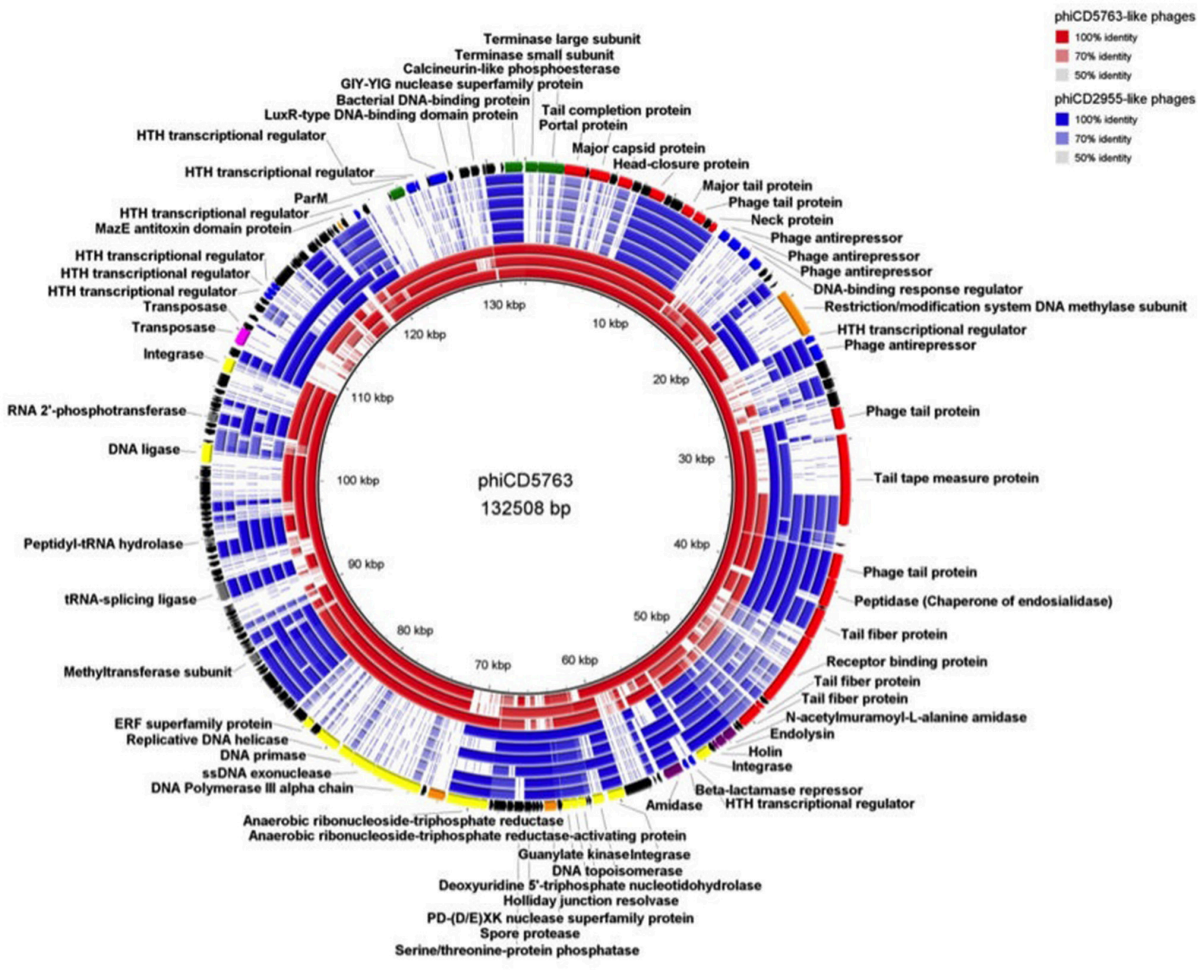
Comparison of the genome of phiCD5763 to those of other phiCD5763-like elements (red) and phiCD2955-like elements (blue). Inner to outer rings: phiCD5774, phiDA00212, phiDA00129, phiCD181, phiF249, phiCDIF1296T, phiP24, and phiDA00128. The predicted CDS of phiCD5763 appear in the outer most ring. The level of transparency of the blocks corresponds to decreasing levels of nucleotide sequence identity (see upper right corner).

**Figure 5:**
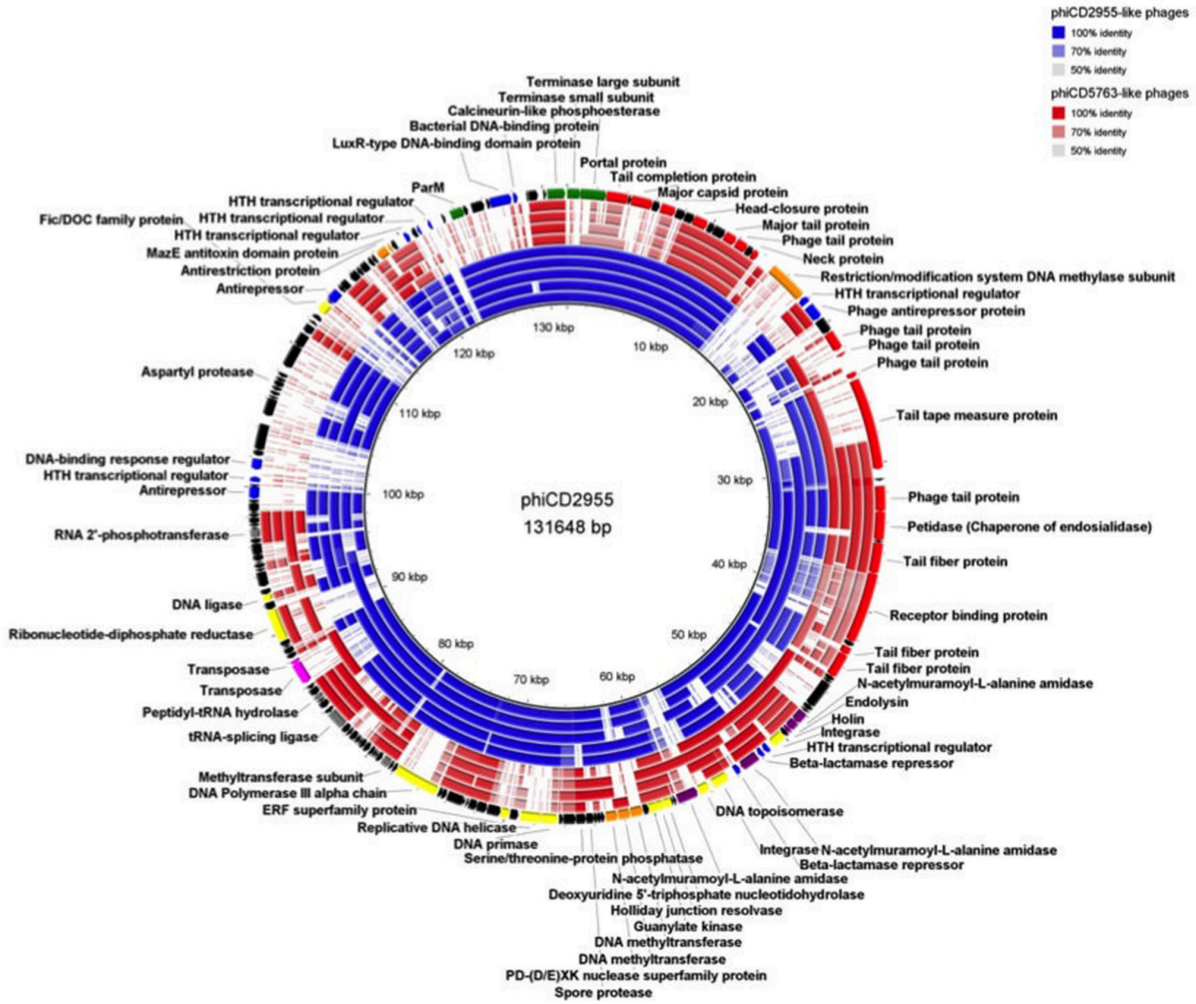
Comparison of the phiCD2955 genome to those of other phiCD2955-like elements (blue) and phiCD5763-like elements (red). Inner to outer rings: phiCD211/phiCDIF1296T, phiF249, phiCD181, phiP24, phiDA00128, phiDA00129, phiDA00212, phiCD5763, and phiCD5774. The predicted CDS of phiCD2955 appear in the outer most ring. The level of transparency of the blocks corresponds to decreasing levels of nucleotide sequence identity (see upper right corner).

Comparison of circular genome maps of reconstructed big phage genomes indicated diversity hotpots (Figures 4, 5). Using phiCD5763 as a reference, the most visible differences between phiCD5763-like and phiCD2955-like phages were concentrated at the 5′-end of their genomes (ORFs 1–8: terminases and structural proteins), the structural-disrupting regulation and host adaptation module (ORFs 17–26), a fragment of the replication-modification-recombination module (ORFs 74–82: replicative proteins and DNA-sulfur modification enzyme), and ORFs 162–169 (*parM* and regulation) (Figure 4). Alternatively, ORFs 1–8 (structural and DNA packaging), 64–71 (replication and other functions), 79 (*pol*α), 134–142 (hypothetical), and 160–169 (*parM* and regulation) emerged as diversity hotspots when phiCD2955 was used as a reference (Figure 5).

### phiCD5763 is a functional phage

Plaque assays of mitomycin C induced LIBA-5763 were carried out against a panel of isolates to determine whether phiCD5763 is an inducible and functional prophage. In this panel of experiments we expected more than one phage to be induced because LIBA-5763 harbors five putative 30–70 kb prophage genomes integrated in its chromosome (four Myoviridae and one Siphoviridae, data not shown), in addition to the extrachromosomal phiCD5763 genome. Large clear plaques were formed on CD630 (RT12/ST54), while medium clear plaques were formed on CD843 (RT103).

**Figure 6:**
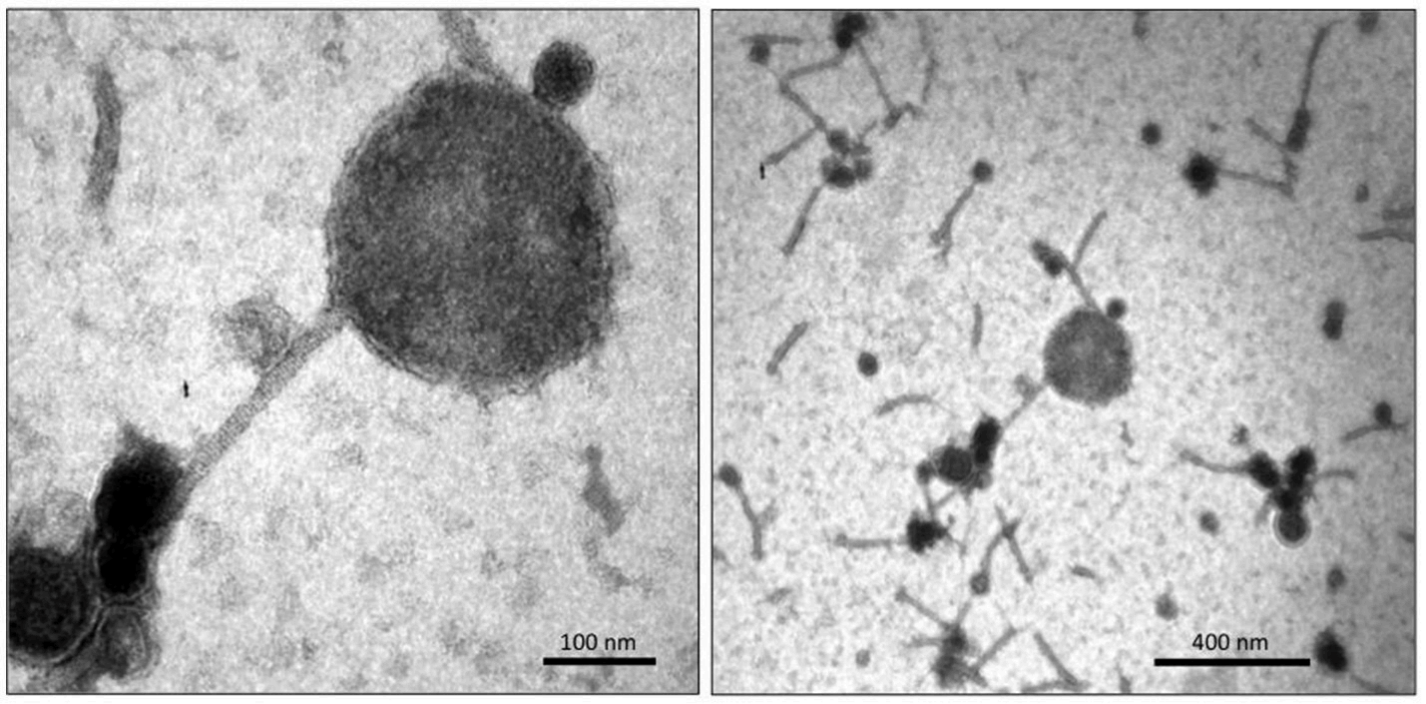
TEM micrographs of phiCD5763/CD630 suspensions showing a big phage particle **(Left)** amongst particles of standard sizes **(Right)**.

Single plaque propagation and subsequent purification of phage suspensions on either CD630 (i.e., phiCD5763/CD630) or CD843 (i.e., phiCD5763/CD843) were examined by TEM. Whereas, the phiCD5763/CD630 phage suspension yielded particles of mixed morphologies (Figure 6), the phiCD5763/CD843 phage suspension yielded homogeneous standard sized particles (data not shown).

**Figure 7:**
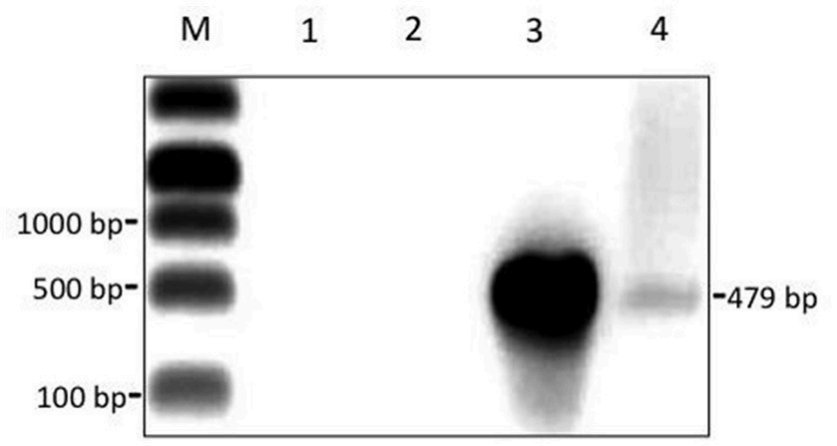
A semi-purified phage suspension obtained through propagation of phiCD5763 in strain CD630 contains the phage gene *dndB*. Gel electrophoresis of PCR products. Lane M, DNA marker; Lane 1, no-template control; Lane 2, genomic DNA from CD630 (negative control); Lane 3, genomic DNA from isolate LIBA-5763 (positive control); Lane 4, DNA isolated from the cell-free phage suspension used for TEM in Figure 6.

Some particles in the phiCD5763/CD630 suspension had large heads 205.6 ± 25.6 nm in diameter (average of five capsids measured in independent samples), which were found infrequently (0.1%) compared to particles of standard size (55–72 nm in diameter) (Figure 6). PCR detection of phiCD5763 DNA was positive in the phiCD5763/CD630 phage suspension and the lysogen LIBA-5763, but not in the indicator host strain CD630 (Figure 7). PCR was also negative for phiCD5763 in the phiCD5763/CD843 suspension.

## Discussion

*C. difficile* NAP_CR1_/RT012/ST-54 isolates caused an epidemic in Costa Rica and are now widely distributed in hospitals from this country (López-Ureña et al., 2016). Genome sequence analysis of several of these isolates in an earlier study revealed the presence of prophages that were novel, particularly in genome size and composition compared to other *C. difficile* phage genomes (Ramírez-Vargas et al., 2017). Here, we report the finding of at least two families of big phage genomes among WGS of NAP_CR1_ and non-NAP_CR1_ strains recovered in several countries and demonstrate that a representative of one of these phage families is functional and capable of infecting other Clade 1 strains of *C. difficile*. We anticipate that a third group of big phage exists, but better sequence assemblies are required to corroborate this statement. It is unlikely that this third group of big phage corresponds to sequence artifacts, as we observed them in high-coverage WGS assembled with edena, SPAdes and a5 (coverage 140–300X, data not shown) and more recently in a Illumina and PacBio sequences for three Clade C-I isolates from Costa Rica (unpublished results). The correspondence of the proteome-based phylogeny and the phylogenetic reconstruction based on the *terLS, parM*, and *polA* genes turn these four genes into appropriate phylogenetic markers for *C. difficile* big phage.

The conserved sequence of big phages derived from NAP_CR1_ isolates recovered 9 years apart, along with the presence of circular contigs in WGS assemblies, strongly suggest the phages do not integrate into the chromosomes of their bacterial hosts. Circular extrachromosomal DNA molecules of viral origin are not rare, but rather difficult to identify (Casjens, 2003). These “plasmidial” phage forms are favored by phage undergoing lysogeny in the environment (Deutsch et al., 2016) and spore-converting phage (Gillis and Mahillon, 2014). In *C. difficile*, a Sipho-phage existing as a circular plasmid with a putative ParA segregation/plasmid maintenance protein is known (Sekulovic et al., 2011). Our big phages are indeed plasmidial but seem to have *parM*-like genes, similar to the prophage CGP3 of *Corynebacterium glutamicum* strain ATCC 13032 (187 kb, Donovan et al., 2015). These actin-based transport systems restrict phage replication to specific subcellular localizations for increased efficiency and allow for intrinsic intracellular movement (Donovan et al., 2015). Putative proteins for addiction mechanisms were also identified in our big phage genomes, supporting their extrachromosomal existence.

The high number of predicted genes for DNA regulation, metabolism, and modification in the reconstructed genomes suggest complex cis-regulation of phage genes and trans-regulation of host genes or other prophage genes within the host chromosome. In this regard, phage antirepressors can act on noncognate repressors and coordinate induction of prophages with unequal induction responses (Lemire et al., 2011). Polylysogenic phages have been shown to regulate one another in complex ways (Matos et al., 2013), and this may apply for the phiCD5763-like phages, as the chromosomes of all NAP_CR1_ isolates carry four different putative Myoviridae prophages (data not shown). Congruently, two of the large phage genomes described here include abundant repressors and CRISPR-associated protein nucleases (e.g., Cas3), which could confer host immunity to superinfection by other phages (Berngruber et al., 2010; Hochstrasser et al., 2014; Bondy-Denomy et al., 2016).

Presence of III RNR genes in the large phage genomes is consistent with the finding that phage RNR distribution correlates with host oxygen requirements (Dwivedi et al., 2013). However, it is interesting to find some phages with class II RNR genes, and may point to evolutionary events involving facultative aerobes and/or facultative anaerobes, where class II RNR genes are usually found. Phage carriage of class II and III genes were mutually exclusive as reported previously (Dwivedi et al., 2013). Organization of the class III RNR genes (*nrdDG*) suggest they are functional, and could provide a fitness advantage for the infecting phage and/or the infected bacterial host (Dwivedi et al., 2013).

Three features of the big phage genomes suggest roles in quorum sensing processes and virulence modulation: (i) a predicted protein with a LuxR-type DNA-binding HTH domain for signaling via LuxS (Hargreaves et al., 2014), which can induce phages (Ghosh et al., 2009); (ii) a predicted spore germination protease needed for *de novo* protein synthesis during spore outgrowth (Wetzel and Fischer, 2015) that may affect host germination kinetics hence transmission; and (iii) HTH transcriptional regulators that may modulate toxin production, such as RepR in phiCD119 (Govind et al., 2009). Although phiCD2955-like elements were found in non-toxigenic *C. difficile* strains, it is possible for these phages to affect virulence upon host acquisition of PaLoc by conjugation (Brouwer et al., 2013).

A number of R-type bacteriocins that kill *C. difficile* have been described (Gebhart et al., 2012, 2015). These so called diffocins contain contractile myophage-like sheath structures coupled to RBPs, which serve as targeting proteins by binding receptors on the surface of a target bacterium. We postulate that the big phage addressed here carry RBPs, as do other Siphoviridae members infecting Firmicutes (Tremblay et al., 2006). Overall, RBPs of the big phages clustered separately from RBPs of CD630 and R20291, and were more related to that of CD4. Some RBPs of phiCD2955-like and phiCD5763-like phages were divergent to RBPs of its own group members. This finding could be an indication of an ancient interaction between both types of big bacteriophages or a highly divergent evolutionary process.

We showed that the 132 kb element phiCD5763 is a functional big bacteriophage that can infect and propagate in CD630. The morphology of phiCD5763 corresponds to that of a siphovirus, and this observation is supported by the lack of genes encoding tail sheet proteins, which are characteristic of Myoviridae phages. The genomes of phiCD211/phiCDIF1296T, phiCD5774, phiCD2955, and other big phage genomes reconstructed in this study also lack genes encoding tail sheet proteins, suggesting they are all big siphoviruses. Despite single plaque propagation of phiCD5763 in CD630, a mixture of medium sized siphovirus particles were observed with phiCD5763 particles. These medium sized siphovirus particles likely originated from LIBA-5763, which harbors four Myoviridae prophages and a Siphovirus prophage of 70 kb. Though not the focus of this study, this medium prophage was only found among NAP_CR1_ isolates from the 487 SmaI macrorestriction pattern. The medium siphovirus particles were unlikely to have originated from the propagating strain CD630, as it harbors only two medium sized Myoviruses (Fortier and Moineau, 2007; Goh et al., 2007).

The capsid of phiCD5763 appears larger than that of *Bacillus megaterium* phage G, which at 160 nm with a genome of ~500 kb is the largest giant phage known so far (Drulis-Kawa et al., 2014). This is unexpected for a relatively small genome of 132 kb compared to giant phage genomes of other bacterial species double in length and number of predicted ORFs. We were unable to isolate the big *C. difficile* phage virions by single-plaque propagation for in depth analysis due to low numbers, possibly because the large phage particles had difficulty passing through 0.22 μm filters used for purification. It is also possible that the presence of other prophages in LIBA-5763 regulated the production of phiCD5763, as reported for multi-lysogenic phages of *Enterococcus faecalis* (Matos et al., 2013).

Through comparative genomics we found various types of extrachromosomal elements of phage origin in 32 NAP_CR1_ isolates and 177 *C. difficile* isolates from different MLST types, sources, time points, and countries. A detailed analysis of reconstructed circular genome sequences and phiCD211/phiCDIF1296T confirmed that although their overall genome synteny is conserved, they differ radically in nucleotide sequence identity, suggesting rapid evolution. Furthermore, through phage infection assays, electron microscopy, and PCR we provide evidence that one of these prophages is functional in replication and infection of other MLST Clade 1 strains of *C. difficile*. Whole genome sequencing of phiCD5763 purified by CsCl density gradients can be used to confirm our findings and to determine whether there is DNA heterogenicity during packaging of the big phage.

Our results indicate that the relationship between *C. difficile* and different variants of big bacteriophages is more widespread than it has been realized. The biology behind this extensive interaction is still unclear, although it is known that phage can trigger phenotypic conversions that influence the virulence of their bacterial hosts (Łoś and Wegrzyn, 2012), lateral gene transfer processes (Canchaya et al., 2003), and the structure of the human gut microbiota (Ventura et al., 2011). New knowledge on *C. difficile* phage is relevant not only because it extends our current understanding of the biology of this emerging human pathogen, but also because phage can be exploited as genetic tools or applied as novel therapeutics.

## Author contributions

GR-V: performed bioinformatics analyses and the PCR experiments, prepared samples for EM, and helped draft the manuscript; SG: propagated phiCD5763, generated phage suspensions, and edited the manuscript; CR: conceived the study, performed some bioinformatics analyses, obtained funding, and wrote the manuscript.

### Conflict of interest statement

The authors declare that the research was conducted in the absence of any commercial or financial relationships that could be construed as a potential conflict of interest.
