# Supplementary material for: The Novel Phages phiCD5763 and phiCD2955 Represent Two Groups of Big Plasmidial Siphoviridae Phages of Clostridium difficile
Source: Front Microbiol. 2018 Jan 22;9:26. doi: 10.3389/fmicb.2018.00026 (PMC5786514; doi:10.3389/fmicb.2018.00026)
Supplement: Supplementary file 2 [file Table2.DOCX]

| Isolate | Ribotype | Origin | Reference (DOI) |
| --- | --- | --- | --- |
| CD37 | 009 | Human | 10.4161/mge.19297 |
| CD062 | 010 | Human | 10.1128/AEM.71.2.1079-1083.2005, 10.1128/mBio.00840-13 |
| CD80 | 010 | Human | 10.1128/mBio.00840-13 |
| CD630 | 012 | Human | 10.1038/ng1830 |
| CD843 | ND* | Human | 10.1099/mic.0.2006/002436-0 |
| WAP55 | UK 237 | Porcine | Unpublished |

Supplementary Table 2. *Clostridium difficile* isolates used for plaque assays of mitomycin C-induced *C. difficile* LIBA-5763

*Not determined
