# Supplementary material for: The Novel Phages phiCD5763 and phiCD2955 Represent Two Groups of Big Plasmidial Siphoviridae Phages of Clostridium difficile
Source: Front Microbiol. 2018 Jan 22;9:26. doi: 10.3389/fmicb.2018.00026 (PMC5786514; doi:10.3389/fmicb.2018.00026)
Supplement: Supplementary file 4 [file Table4.DOCX]

Supplementary Table 4. *C. difficile* phages of medium genome size included in the Phamerator analyses

| *C. difficile* phage | Host strain | Size (kb) / No ORF | Family | Accession number |
| --- | --- | --- | --- | --- |
| phiMMP04 | CD380 | 31.6 / 50 | Myoviridae | NC_019422 |
| phiCDHM11 | Unknown | 32.0 / 48 |  | NC_029001 |
| phiCDHM14 | Unknown | 32.6 / 50 |  | LK985321 |
| phiCD481-1 | Unknown | 32.8 / 51 |  | LN681538 |
| phiCD506 | Unknown | 33.2 / 52 |  | LN681540 |
| phiCDHM13 | Unknown | 33.5 / 50 |  | NC_029116 |
| phiMMP01 | Unknown | 44.4 / 77 |  | LN681541 |
| phiMMP02 | CD343 | 48.3 / 76 |  | NC_019421 |
| phiCD505 | Unknown | 49.3 / 72 |  | LN681539 |
| phiCDKM9 | Unknown | 49.8 / 75 |  | KX228399 |
| phiCD27 | Unknown | 50.9 / 75 |  | NC_011398 |
| phiMMP03 | CD368 | 52.2 / 87 |  | LN681542 |
| phiCD119 | CD602 | 53.3 / 79 |  | NC_007917 |
| phiCDHM1 | CD105HS6 | 54.2 / 84 |  | NC_024144 |
| phiCDHM19 | Unknown | 54.2 / 88 |  | NC_028996 |
| phiC2 | CD242 | 56.5 / 85 |  | NC_009231 |
|  |  |  |  |  |
| phiCD6356 | DPC6359 | 37.6 / 59 | Siphoviridae | NC_015262 |
| phiCD38-2 | CD274 | 41.0 / 55 |  | NC_015568 |
| phiCD111 | Unknown | 41.5 / 53 |  | LN681535 |
| phiCD146 | Unknown | 41.5 / 52 |  | LN681536 |
| phiCD24-1 | Unknown | 44.1 / 57 |  | LN681534 |
|  |  |  |  |  |
| phiSemix9P1 | Semix9 | 56.6 / 74 | Unknown | KX905163 |
