# Supplementary material for: The Novel Phages phiCD5763 and phiCD2955 Represent Two Groups of Big Plasmidial Siphoviridae Phages of Clostridium difficile
Source: Front Microbiol. 2018 Jan 22;9:26. doi: 10.3389/fmicb.2018.00026 (PMC5786514; doi:10.3389/fmicb.2018.00026)
Supplement: Supplementary file 11 [file Image1.PDF]

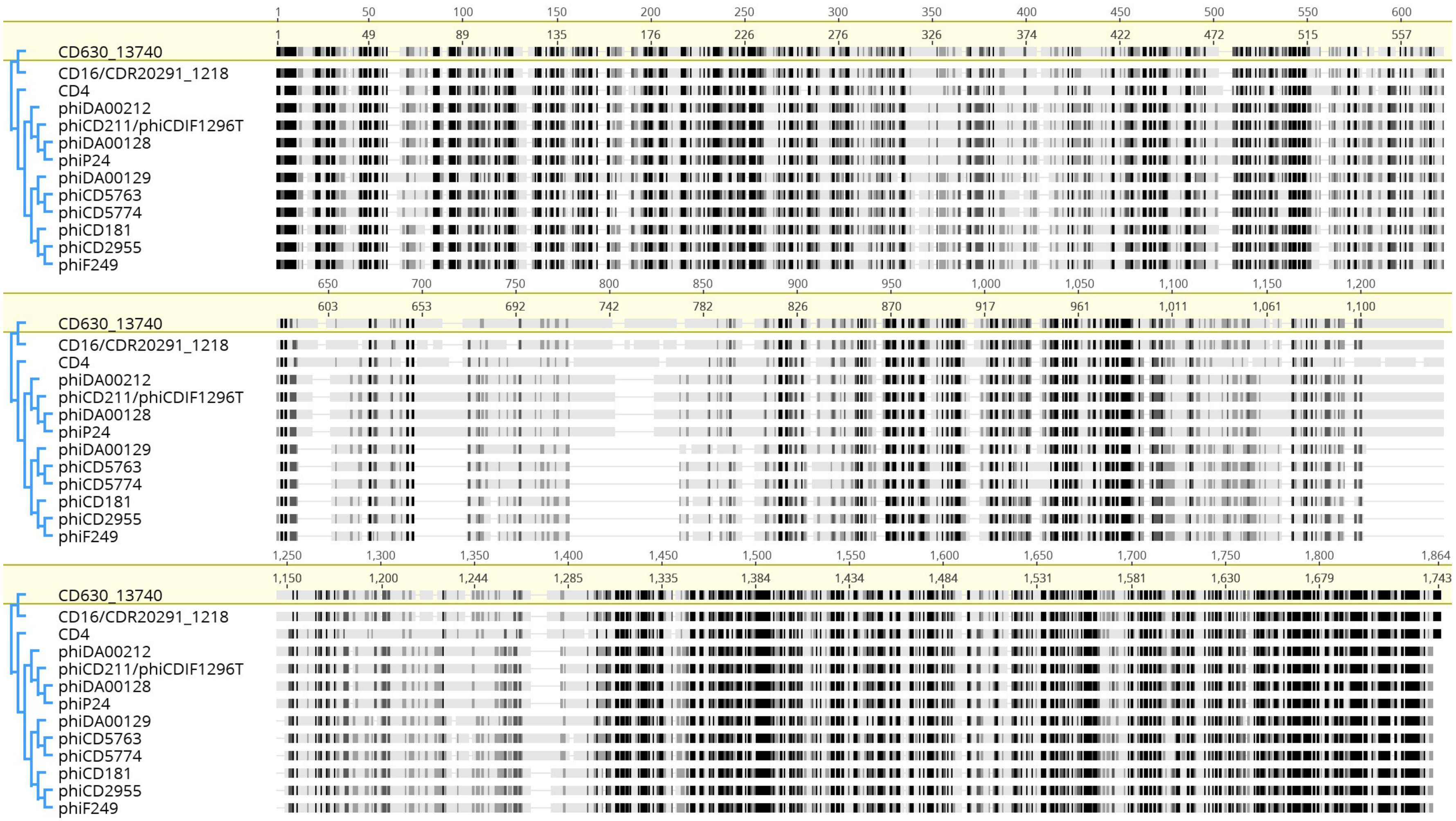

Supplementary Figure 1. Alignment of the sequences of diffocin receptor binding proteins (RBPs) of *C. difficile* CD630 (CD630_13740), R20291 and C16 (CD16/CDR20291_1218), and CD14, and predicted RBPs in phiCD5763-like genomes (phiDA00212, phiDA00129, phiCD5763 and phiCD5774) and phiCD2955-like genomes (phiCD211/ phiCDIF1296T, phiDA00128, phiP24, phiCD181, phiCD2955, and phiF249). The alignment includes 1864 amino acid residues. The sequence of CD630_13740 (1743 residues) was selected as a reference. Identical residues are highlighted in black and the intensity of grey blocks indicates similarity. Sequence gaps are shown with horizontal thin lines.
